# School nutritional capacity, resources and practices are associated with availability of food/beverage items in schools

**DOI:** 10.1186/1479-5868-10-26

**Published:** 2013-02-19

**Authors:** Louise C Mâsse, Judith E de Niet

**Affiliations:** 1 University of British Columbia, School of Population and Public Health, F508-4480 Oak Street, Vancouver, BC V6H 3V4, Canada

**Keywords:** Nutrition, School environment, Organizational support, School policy, Availability of food, Availability of sweetened beverages, Fruit, Vegetables, Junk food, Less healthful food

## Abstract

**Background:**

The school food environment is important to target as less healthful food and beverages are widely available at schools. This study examined whether the availability of specific food/beverage items was associated with a number of school environmental factors.

**Methods:**

Principals from elementary (n = 369) and middle/high schools (n = 118) in British Columbia (BC), Canada completed a survey measuring characteristics of the school environment. Our measurement framework integrated constructs from the Theories of Organizational Change and elements from Stillman’s Tobacco Policy Framework adapted for obesity prevention. Our measurement framework included assessment of policy institutionalization of nutritional guidelines at the district and school levels, climate, nutritional capacity and resources (nutritional resources and participation in nutritional programs), nutritional practices, and school community support for enacting stricter nutritional guidelines. We used hierarchical mixed-effects logistic regression analyses to examine associations with the availability of fruit, vegetables, pizza/hamburgers/hot dogs, chocolate candy, sugar-sweetened beverages, and french fried potatoes.

**Results:**

In elementary schools, fruit and vegetable availability was more likely among schools that have more nutritional resources (OR = 6.74 and 5.23, respectively). In addition, fruit availability in elementary schools was highest in schools that participated in the BC School Fruit and Vegetable Nutritional Program and the BC Milk program (OR = 4.54 and OR = 3.05, respectively). In middle/high schools, having more nutritional resources was associated with vegetable availability only (OR = 5.78). Finally, middle/high schools that have healthier nutritional practices (i.e., which align with upcoming provincial/state guidelines) were less likely to have the following food/beverage items available at school: chocolate candy (OR = .80) and sugar-sweetened beverages (OR = .76).

**Conclusions:**

School nutritional capacity, resources, and practices were associated with the availability of specific food/beverage items in BC public schools. Policies targeting the school environment are increasingly being considered as one of the strategies used to address childhood obesity, as a result it is important to further understand the factors associated with the availability of specific food/beverage items at school.

## Background

Overwhelming evidence demonstrates a worldwide increase in childhood obesity [1] and Canada is no exception to this trend [1]. In 2004, one quarter (26%) of Canadian children aged 2 to 17 years were either overweight or obese, representing a sharp increase from a combined prevalence of 15% in 1978/1979 [1]. While obesity is a complex and multi-factorial problem, many have suggested focusing on the school food environment as part of a comprehensive multi-setting approach to address childhood obesity [2].

The availability of less healthful food and beverages in schools is widespread [3-5]. Despite recent changes to improve the school food environment, the availability of some high fat food such as pizza and hamburgers, remains alarmingly high in U.S. schools (73.9% and 82.6%, respectively for elementary and middle/ high schools) [5]. Similar to U.S. schools, Canadian elementary schools have fewer vending machines; however, less healthful food and beverages are widely available to all grades as they are made available through other outlets (e.g., cafeteria, school stores) [3].

The school food environment has been shown to influence student eating behaviors [6-12]. In schools where less healthful food and beverages are widely available, students have higher intake of these items, consume fewer fruit and vegetables, and have higher total fat intake [7-11]. In addition, increasing the availability of healthful food and beverages in schools has been associated with improved dietary intake [13,14]. Although evidence linking the school food environment with student Body Mass Index (BMI) is mixed [6], both the frequency at which fruit and vegetables are available and the availability of less healthful food in vending machines or other venues at school have been associated with higher BMI in students [14]. As the school food environment may influence the eating behaviors of children in that context, it is important to understand the factors associated with the availability of specific food/beverage items at schools.

The school food environment may be influenced by several factors, such as policies/guidelines that limit the availability of certain food/beverages items, restricting the use of food as rewards in the classroom, setting standards for nutrition education, restricting certain marketing practices, having adequate resources and capacity at the school and district levels, having a supportive school community, having access to nutritional expertise locally, having access to nutritional programs that promote healthy eating, and having a favorable socio-demographic profile [15-22]. With the exception of school food policies/guidelines, where emerging data links policies with students’ dietary intake and BMI [18,23-27], the extent to which other school factors are related to the availability of specific food/beverage items at school has received little attention. However, uptake of nutrition and health programs in the school setting have been found to be highest in schools that have more supportive policies; better organizational climate, capacities and resources; and have more support from school principals [28-30]. This suggests that these environmental factors might be associated with the availability of specific food/beverage items in the school setting. Therefore, the purpose of this study was to determine whether the availability of fruit, vegetables, pizza/hamburgers/hot dogs, chocolate candy, sugar-sweetened beverages, and french fried potatoes was associated with a number of school environmental factors: (1) policy institutionalization of nutritional guidelines at the district and school levels, (2) climate, (3) nutritional capacity and resources, (4) nutritional practices at school, and (5) school community support for enacting stricter nutritional guidelines at school. These associations were examined separately for elementary schools and middle/high schools. Differences were expected given that food and beverages are more likely available in higher grades [5,31].

Note we examined these associations in schools located in British Columbia (BC), Canada which has a markedly different school food environment than other countries. Unlike other countries, Canada does not have a federally subsidized school meal program [32]. Some school districts, through funding from the Ministry of Education, enable schools to offer school meal programs to vulnerable students (breakfast program, hot lunch program, bag lunch program, or snack program). School meal programs are often managed through partnerships and donations schools/districts have negotiated with external agencies. As many schools do not have on-site cooking facilities, schools can contract food vendors to prepare the school meals following the non-mandated guidelines published by the province/state for administering the program. In addition, in our context the availability of permanent food outlets is much lower in elementary schools than in middle/high schools (45% versus 95% have permanent food outlets, respectively); however, 82% of elementary schools have external vendors contracted to bring in A La Carte lunch options (e.g., pizza, hamburgers, hot dogs), varying from multiple times a week to a few times per month [3]. Importantly, food and beverages made available or sold to students were not mandated to meet any nutritional guidelines until the 2008/2009 school year, as the province/state enacted guidelines to support healthy eating at schools which aligned with the 2007 Canada Food Guide [33].

## Methods

### Participants

Public school principals in BC were targeted for this study. Given the relatively small number of Francophone, Independent, First Nations, and alternative schools in BC, principals of these schools were excluded as this study could not address their specific issues of these schools. Study approval was received in 43 of the 59 school districts (73% response rate); however, three districts were excluded as they were participating in another study conducted by the same research team. Of the 1067 eligible principals, 513 principals completed the school environment survey (48% response rate). Respondents from schools that included all grades (i.e., elementary, middle, and high schools n = 13) or had less than 50 students (n = 13) were excluded. In total, 369 elementary schools and 118 middle/high schools from 38 districts provided data for the analyses.

### Procedures

This study was approved by the University of British Columbia Research Ethics Board. Prior to data collection, school district approval was obtained. In January of 2008, principals received a package that included an invitational letter, consent form, a hard copy of the school environment survey (which took 30 minutes to complete), and a pre-paid self return envelope. Approximately two weeks after the invitational package was mailed, a research staff member contacted the principals to determine if they had specific questions about the study. Principals who did not return the school environment survey received a second mailing. If completed surveys were not received within a 3-week period, principals received a reminder email with a link to the online survey as an alternative way of completing the survey. Principals were encouraged to seek out the expertise of their staff if assistance was needed for filling out sections of the survey (e.g., the nutrition environment section). Data collection ended in June 2008. Principals received a $10 gift card for completing the survey.

### Measures

**Figure 1:**
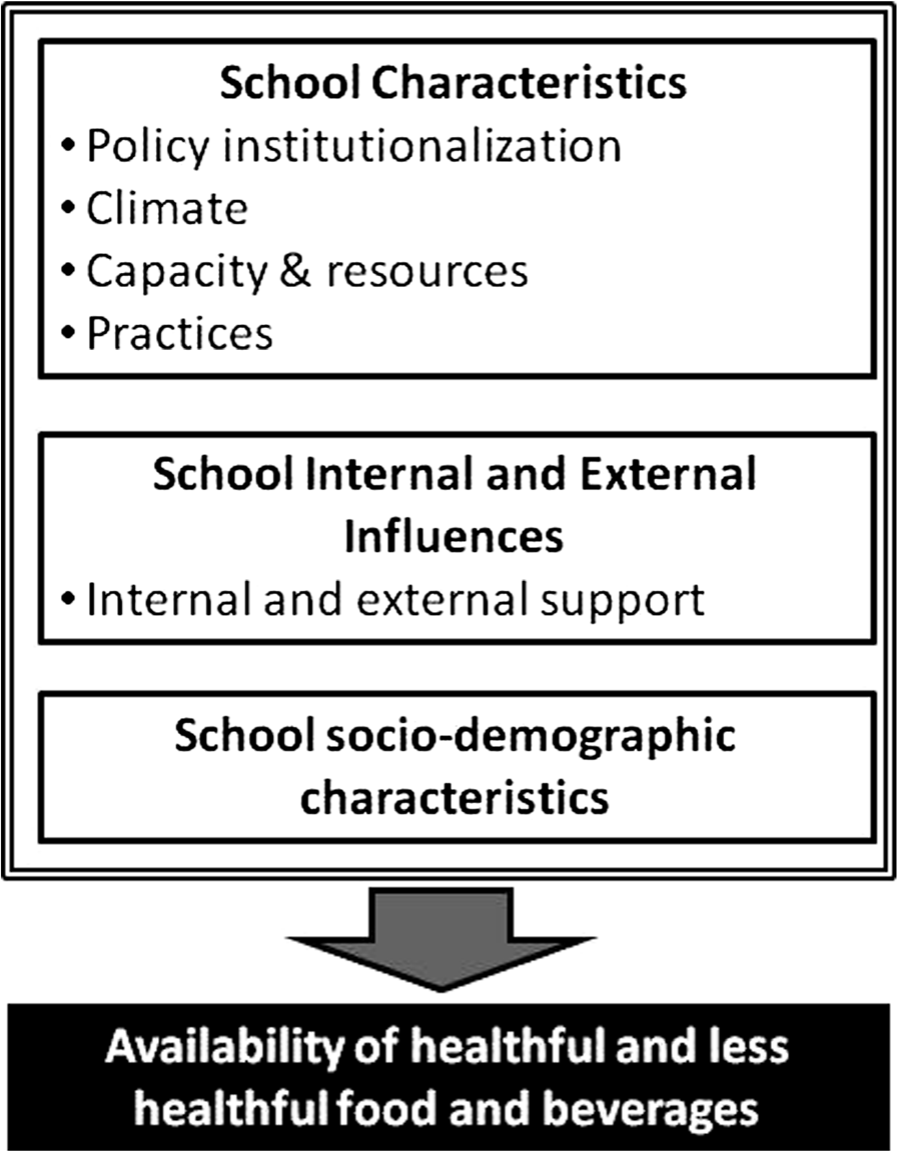
School environmental factors hypothesized to be associated with availability of specific food/beverage items.

Operationalization of the measures integrates central constructs from the Theories of Organizational Change and elements of Stillman’s Tobacco Policy Framework adapted for the context of this study (see Figure 1). From the Theories of Organizational Change, our assessment framework incorporates assessment of policy institutionalization (e.g., school and district policies, guidelines, or requirements) as well as measures of organizational climate, capacity and resources, and practices to support healthy eating and nutrition education at schools. From Stillman’s Tobacco Policy Framework [34], our measurement framework includes assessment of internal and external influences that may impede or facilitate implementation of healthy eating practices or policies at school. Whenever possible, existing measures were used to assess the constructs of interests. Content review of our measures was conducted by having relevant provincial Ministry staff and school principals review the relevance of the items in the context of BC. A description of the measures follows as well as a description of the psychometric properties of the scales used to measure these constructs.

### School characteristics (independent variables)

Policy institutionalization measured the extent to which districts and schools had nutritional policies/guidelines/requirements related to the availability of food and beverages, qualifications of food personnel, and nutrition education at school. Two scales assessed policy institutionalization: [1] District guidelines is a 3-item scale that assessed whether principals perceived their district’s guidelines to be average, above average, or below average compared to other districts with respect to food and beverages sold or made available to students, staffing requirements for school food personnel, and nutrition education requirements; and [2] School guidelines is a 7-item scale that measured whether schools have guidelines in place that ban food advertizing, prohibit use of less healthful food as reward, require healthier food choices be subsidized, provide educational requirements for the school food personnel, and include requirements for nutrition education. Response options for these items included “no”, “in the process of developing guidelines”, and “yes”. These options were dichotomized for analysis purposes (“0” for “no” and “in the process” and “1” for “yes”). In addition, the scale included two items that measured whether the school incorporates the Canada’s Food Guide into the curriculum with “yes” and “no” as response options.

Climate assessment was derived from Hoy’s school climate measure [35-38]. The scale, using seven response options, includes 10 semantic differential items that measured whether the overall school climate is collegial, supportive, conciliatory, friendly, warm, open, welcoming, accepting of change, accommodating, and trusting.

Capacity and resources measured two dimensions: nutritional resources and program participation. Nutritional resources is a 5-item scale based on Hoy’s organizational Health Inventory of elementary schools [39]. The scale assessed whether principals perceived their school’s nutritional resources to be average, above average, or below average compared to other schools with respect to the number of staff involved in food preparation and management, eating facility, access to a local nutritionist, access to caterers and vendors that offer healthier food options, and opportunities to make healthy food choices at school. Program participation was measured with two items: one assessing participation in the BC Milk Program and the other assessing participation in the BC School Fruit and Vegetable Nutritional Program (BCSFVNP), with “yes” and “no” as response options. The BC Milk program subsidizes the costs of milk to students in K-12 and in elementary schools the milk can be delivered in the classroom instead of the cafeteria. The BCSFVNP was launched in 2005 and provides K-12 schools with 14 free deliveries of at least two servings of locally grown fresh fruit and/or vegetables for every student throughout the school year. Both programs are run by school volunteers.

School nutrition practices is a 3-item index that assessed whether current school practices are aligned with next school years’ upcoming mandated provincial/state guidelines that eliminate the availability of less healthful food and beverages in schools and follows the 2007 Canada’s Food Guide eating recommendations [33]. Specifically, this index assessed whether schools implemented the guidelines for food and beverages sold or made available to students in the following locations: snack bar/school store(s); vending machine(s); and cafeteria. Response options for these items included “no”, “in the process of developing requirements”, and “yes”. These options were dichotomized for analysis purposes (“0” for “no” and “in the process” and “1’ for “yes”) and averaged across the three items.

### School internal and external influences (independent variables)

Internal and external support is a 7-item scale that assessed perceived support from parents, staff, students, and the larger community for eliminating less healthful food and beverages in schools as well as assessing whether principals believed schools can play a role in addressing childhood obesity. All items were measured on a 4-point Likert-scale (strongly disagree to strongly agree).

### School socio-demographic characteristics (covariates)

School size (total number of students) and school setting (categorized as inner city/urban, suburban, or rural) were included in the analyses as covariates.

### Availability of specific food/beverage items (dependent variables)

The School Health Policies and Programs Study (SHPPS) questions were used to assess availability of specific food/beverage items at school for lunch in a typical week [40]. The availability of the following food/beverage items were measured: [1] fruit; [2] vegetables; [3] pizza/hamburgers/hot dogs; [4] chocolate candy; [5] sugar-sweetened beverages (e.g., pop, iced tea, sport drinks or fruit drinks that are not 100% fruit juice); and [6] french fried potatoes.

### Psychometric properties of the measures

The psychometric properties of the scales were assessed in the larger sample using exploratory factor analysis. We extracted a 5-factor solution using the principal component extraction method with a promax rotation. Results from the factor analysis showed that a 5-factor solution replicated the hypothesized factor structure with the exception of two items: one item (the internal and external support item that measured whether principals believed schools can play a role in addressing childhood obesity) cross-loaded on two factors (internal and external support and policy institutionalization – school guidelines); and another item had a low factor loading (.24) (the policy institutionalization – school guidelines item that measured requirements for nutrition education in schools). The 5-factor solution explained 58% of the total variance and each scale had adequate internal consistency (Cronbach alpha (α)), although one was slightly less than the acceptable cut-off of .70 (Policy institutionalization – district guidelines α = .79 with factor loadings ranging from .78 to .89; Policy institutionalization – school guidelines α = .64 with factor loadings ranging from .50 to .68 (except one item had a loading of .24); Climate α = .94 with factor loadings ranging from .63 to .88; Capacity & resources – nutritional resources α = .72 with factor loadings ranging from .62 to .73; and Internal and external support α = .72 with factor loadings ranging from .30 to .73). Correlations among factors were low (less than .30).

### Statistical analyses

All analyses were conducted using the STATA software version 11.1 (StataCorp, Texas USA). Nine hierarchical mixed-effects logistic regressions (xtmelogit) were employed to address the research questions. To account for the nested structure of the data, the district code was entered in the analyses. Multivariate associations were examined by including the covariates and all the independent variables in the model. Separate analyses were conducted for elementary schools and middle/high schools. Analyses for the elementary schools were restricted to food and beverages with more than 10% availability. To account for multiple comparisons, significance level was set at a stringent alpha level of .01 and trends toward significance were set at an alpha level of .05.

Missing data were imputed using multiple imputation techniques (missing data ranging from 0.6 to 15.8%). All missing data were imputed on the raw data using STATA’s Expectation Maximization method which assumes the data is missing at random and unrelated to the outcome [41]. The dependent variables were included in the imputation model but only missing data on the covariates and independent variables were imputed. A total of five imputations were used.

## Results

### School environmental factors

Participating middle/high schools were significantly larger than the elementary schools (Table 1). Schools located in urban, suburban, and rural areas were well represented. Four of the six school characteristics differed significantly (p < .01) between elementary and middle/high schools (Table 1). Specifically, elementary schools had a better climate, less nutritional resources, higher participation in nutritional programs (BC Milk Program and the BCSFVNP), and better nutritional practices which align with upcoming provincial/state nutritional guidelines. In addition, elementary schools had significantly more support (internal and external) for enacting stricter nutritional guidelines at school than did middle/high schools.

**Table 1:** Descriptive information about the elementary (N = 366) and middle/high (N = 116) schools that participated in the study

		**Elementary schools**	**Middle/high schools**	**Group comparisons**
		% or mean (SD)	% or mean (SD)	χ^2^ or t-test
		[range]	[range]	p-value
Number of schools per district		9.7	3.1	
		[1 - 38]	[1 – 11]	
**School socio-demographic characteristics**				
School size		283 (146)	838 (424)	t(1) = 21.44
(N_elementary schools_ (N_e_) = 379;N_middle/high schools_ (N_mh_) = 117)		[50 - 1062]	[121 - 2100]	p < 0.001**
School setting (N_e_ = 361;N_mh_ = 116)	Urban	36.8%	31.9%	χ^2^ (1) = 0.98
	Suburban	35.2%	37.1%	p = 0.614
	Rural	28.0%	31.0%	
**School characteristics**				
Policy institutionalization – District guidelines		2.1 (0.4)	2.1 (0.4)	t(1) = 1.01
(N_e_ = 314; N_mh_ = 102)		[1.0 – 3.0]	[1.0 – 3.0]	p = 0.312
Policy institutionalization – School guidelines		0.4 (0.3)	0.4 (0.2)	t(1) = 0.29
(N_e_ = 254; N_mh_ = 89)		[0.0 – 1.0]	[0.0 – 1.0]	p = 0.773
Climate		2.4 (0.5)	2.2 (0.6)	t(1) = -3.00
(N_e_ = 352;N_mh_ = 113)		[1.0 – 3.0]	[1.0 – 3.0]	p = 0.003**
Capacity & resources – Nutritional resources		1.8 (0.4)	2.0 (0.5)	t(1) = 4.64
(N_e_ = 306; N_mh_ = 104)		[1.0 – 2.8]	[1.0 – 3.0]	p < 0.001**
Capacity & resources – Program participation (N_e_ = 335; N_mh_ = 93)	BCSFVNP (% yes)	43.3%	25.8%	χ^2^ (1) = 9.31
				p = 0.002**
	BC Milk Program (% yes)	38.2%	25.8%	χ^2^ (1) = 4.89
				p = 0.027*
School nutrition practices		7.4 (2.1)	5.6 (4.1)	t(1) = -6.26
(N_e_ = 359;N_mh_ = 117)		[0 – 10]	[0 – 10]	p < 0.001**
**School internal and external influences**				
Internal and external support		2.8 (0.3)	2.7 (0.4)	t(1) = -2.59
(N_e_ = 273; N_mh_ = 104)		[1.9 – 3.9]	[1.7 – 3.9]	p = 0.010*

BCFVNP = British Columbia Fruit and Vegetable Nutritional Program; BC = British Columbia.

** Significant at p < .01.

* Trend towards statistical significance at p < .05.

### Availability of food and beverages

The availability of fruit, vegetables, and pizza/hamburgers/hot dogs was significantly lower in elementary schools compared to middle/high schools (Table 2). Chocolate candy, french-fried potatoes, and sugar-sweetened beverages were either not available or rarely available in elementary schools than compared to middle/high schools.

**Table 2:** Percent of elementary (N = 369) and middle/high schools (N = 118) reporting availability of specific food/beverage items

**Availability of**	**Elementary schools**	**Middle/high schools**	**Group comparisons** χ^2^ p-value
Fruit	27.9%	74.6%	χ^2^ (1) = 81.67 p < 0.001**
Vegetables	9.2%	69.5%	χ^2^ (1) = 179.03 p < 0.001**
Pizza, hamburgers, or hot dogs	13.8%	63.6%	χ^2^ (1) = 115.33 p < 0.001**
Sugar-sweetened beverages (pop, iced tea, sport drinks or fruit drinks that are not 100% fruit juice)	5.4%	62.7%	χ^2^ (1) = 188.41 p < 0.001**
French fried potatoes	0%	41.5%	χ^2^ (1) = 170.37 p < 0.001**
Chocolate candy	0%	37.3%	χ^2^ (1) = 151.26 p < 0.001**

** Significant at p < .01

* Trend towards statistical significance at p < .05.

### School environmental factors associated with availability of specific food/beverage items

Tables 3 and 4 summarize the results of the hierarchical mixed-effect logistic regression analyses examining which school environmental factors were associated with the availability of specific food/beverage items in elementary and middle/high schools.

**Table 3:** Factors associated with availability of fruit, vegetables and pizza/hamburgers/hot dogs in elementary (N = 369) and middle/high schools (N = 118)

			**Availability of fruit**		**Availability of vegetables**		**Availability of pizza/hamburgers/hot dogs**	
			**Elementary schools**	**Middle/high schools**	**Elementary schools**	**Middle/high schools**	**Elementary schools**	**Middle/high schools**
			Coefficient	Coefficient	Coefficient	Coefficient	Coefficient	Coefficient
			[95% CI]	[95% CI]	[95% CI]	[95% CI]	[95% CI]	[95% CI]
			p-value	p-value	p-value	p-value	p-value	p-value
**Constant**			-2.03	-2.77	-7.76	-1.03	-4.55	3.25
			[-5.32; 1.27]	[-8.33; 2.80]	[-12.70; -2.83]	[-5.43; 3.37]	[-8.42; -0.67]	[-1.42; 7.92]
			p = .055	p = .329	p = .002**	p = .647	p = 022*	p = .170
			**School socio-demographic characteristics (Covariates)**					
School size			-0.79	-0.12	-0.41	0.32	0.01	-0.10
			[-1.61; -0.02]	[-0.57; 0.34]	[-1.44; 0.61]	[-0.11; 0.76]	[-0.79; 0.80]	[-0.48; 0.27]
			p = .055	p = .619	p = .429	p = .143	p = .984	p = .580
School setting	Urban		1 (reference)	1 (reference)	1 (reference)	1 (reference)	1 (reference)	1 (reference)
	Suburban		-0.94	0.87	-0.31	0.41	-0.56	0.23
			[-1.69; -0.18]	[-0.81; 2.55]	[-1.49; 0.87]	[-0.84; 1.66]	[-1.55; 0.43]	[-0.86; 1.31]
			p = .015*	p = .310	p = .607	p = .521	p = .270	p = .682
	Rural		-0.03	0.15	.26	-0.51	0.30	-1.42
			[-0.87; 0.82]	[-1.51; 1.82]	[-1.05; 1.56]	[-1.86; 0.84]	[-0.73; 1.33]	[-2.72; -0.12]
			p = .953	p = .858	p = .701	p = .461	p = .565	p = .032*
			**School characteristics**					
Policy institutionalization	District guidelines		-0.97	-0.08	-0.95	-0.49	0.04	-0.08
			[-2.00; 0.07]	[-2.07; 1.92]	[-2.22; 0.31]	[-2.13; 1.14]	[-0.98; 1.05]	[-1.60; 1.45]
			p = .068	p = .940	p = .139	p = .553	p = .944	p = .920
	School guidelines		0.87	-0.55	-0.03	-0.03	-0.61	1.05
			[-0.24; 1.98]	[-3.53; 2.44]	[-1.57; 1.51]	[-2.46; 2.39]	[-1.88; 0.67]	[-1.48; 3.58]
			p = .124	p = .716	p = .966	p = .978	p = .350	p = .408
Climate			-0.48	0.48	0.07	-0.57	0.35	-0.32
			[-1.04; 0.08]	[-0.42; 1.38]	[-0.71; .86]	[-1.42; 0.27]	[-0.33; 1.03]	[-1.07; 0.42]
			p = .092	p = .292	p = .852	p = .183	p = .316	p = .396
Capacity & resources – Nutritional resources			1.91	1.49	1.65	1.75	0.85	0.09
			[0.99; 2.82]	[-0.14; 3.12]	[0.42; 2.89]	[0.42; 3.09]	[-0.16; 1.87]	[-1.01; 1.19]
			p ≤ .000**	p = .073	p = .009**	p = .010**	p = .098	p = .871
Capacity & resources – Program participation	BCSFVNP	No	1 (reference)	1 (reference)	1 (reference)	1 (reference)	1 (reference)	1 (reference)
		Yes	1.51	-0.35	1.00	-0.20	-0.11	0.75
			[0.89; 2.14]	[-1.78; 1.08]	[0.10; 1.90]	[-1.35; 0.96]	[-0.86; 0.64]	[-0.33; 1.83]
			p ≤ .000**	p = .629	p = .029*	p = .738	p = .773	p = .171
	BC Milk Program	No	1 (reference)	1 (reference)	1 (reference)	1 (reference)	1 (reference)	1 (reference)
		Yes	1.12	1.71	0.52	-0.35	0.08	-0.39
			[0.51; 1.72]	[-0.50; 3.93]	[-0.41; 1.44]	[-1.88; 1.17]	[-0.64; 0.80]	[-1.62; 0.85]
			p ≤ .000**	p = .124	p = .270	p = .636	p = .827	p = .528
School nutrition practices			-0.08	0.03	0.05	-0.01	-0.07	-0.06
			[-0.22; 0.06]	[-0.12; 0.18]	[-0.16; 0.25]	[-0.15; 0.12]	[-0.22; 0.08]	[-0.18; 0.05]
			p = .282	p = .695	p = .649	p = .833	p = .383	p = .292
			**School internal and external influences**					
Internal and external Support			0.21	0.03	1.02	0.08	0.26	-0.54
			[-0.85; 1.26]	[-1.42; 1.48]	[-0.43; 2.47	[-1.16; 1.33]	[-0.84; 1.36]	[-1.66; 0.57]
			p = .700	p = .969	p = .169	p = .896	p = .643	p = .339

CI = Confidence Interval; BCSFVNP = BC School Fruit and Vegetable Nutritional Program; BC = British Columbia;

** Significant at p < .01.

* Trend towards statistical significance at p < .05.

**Table 4:** Factors associated with availability of chocolate candy, sugar-sweetened beverages, and french fried potatoes in middle/high (N = 118) schools

		**Availability of chocolate candy**		**Availability of sugar-sweetened beverages**	**Availability of french fried potatoes**
		Coefficient		Coefficient	Coefficient
		[95% CI]		[95% CI]	[95% CI]
		p-value		p-value	p-value
**Constant**		4.58		-1.11	-0.80
		[-0.75; 9.11]		[-7.17; 4.96]	[-6.82; 5.23]
		p = .092		p = .720	p = .795
**School socio-demographic characteristics (Covariates)**					
School size		0.10		0.28	0.40
		[-0.34; 0.53]		[-0.19; 0.75]	[-0.11; 0.92]
		p = .654		p = .239	p = .123
School setting	Urban	1 (reference)		1 (reference)	1 (reference)
	Suburban	-0.50		0.40	0.14
		[-1.87; 0.87]		[-1.20; 2.00]	[-1.41; 1.68]
		p = .474		p = .620	p = .862
	Rural	-1.51		1.44	-0.30
		[-3.17; 0.15]		[-0.40; 3.28]	[-2.32; 1.72]
		p = .074		p = .126	p = .771
**School characteristics**					
Policy institutionalization	District guidelines	-0.28		-0.77	0.11
		[-2.02; 1.47]		[-2.58; 1.03]	[-1.52; 1.73]
		p = .756		p = .899	p = .400
	School guidelines	0.56		2.05	-0.36
		[-2.18; 3.30]		[-0.63; 4.72]	[-3.26; 2.54]
		p = .687		p = .133	p = .808
Climate		0.03		0.55	-0.23
		[-0.86; 0.92]		[-0.36; 1.45]	[-1.29; 0.82]
		p = .947		p = .234	p = .667
Capacity & resources - Nutritional resources		-0.05		0.19	1.53
		[-1.38; 1.29]		[-1.04; 1.41]	[0.08; 2.98]
		p = .945		p = .766	p = .039*
Capacity & resources - Program participation	BCSFVNP	No	(reference)	1 (reference)	1 (reference)
		Yes	1.02	0.43	0.32
			[-0.71; 2.74]	[-1.02; 1.88]	[-1.28; 1.91]
			p = .234	p = .552	p = .690
	BC Milk Program	No	1 (reference)	1 (reference)	1 (reference)
		Yes	0.19	-0.36	-0.71
			[-1.12; 1.51]	[-1.65; 0.92]	[-2.17; 0.75]
			p = .773	p = .580	p = .343
School nutrition practices			-0.23	-0.27	-0.22
			[-0.37; -0.08]	[-0.42; -0.11]	[-0.40; 0.04]
			p = .002**	p = .001**	p = .019*
**School internal and external influences**					
Internal and external Support			-1.38	-0.28	-0.11
			[-2.84; -0.07]	[-1.75; 1.19]	[-1.80;1.58]
			p = .063	p = .706	p = .901

CI = Confidence Interval; BC = British Columbia; BCSFVNP = BC School Fruit and Vegetable Nutritional Program.

** Significant at p < .01.

* Trend towards statistical significance at p < .05.

### Associations with demographic characteristics

Overall, the school demographic variables were not significantly associated (p < .01) with availability of specific food/beverage items in schools. However, the school setting showed a trend towards statistical significance (p < .05) with fruit availability in elementary schools and with pizza/hamburger/hot dog availability in middle/high schools. Elementary schools located in suburban areas were less likely than those located in urban areas to report fruit availability (Odds Ratio (OR) = 0.39, p = .015). In addition, middle/high schools located in rural areas were less likely than those located in urban areas to report pizza/hamburger/hot dog availability (OR = 0.24, p = .032).

### Associations with school characteristics

In elementary schools, fruit and vegetable availability was more likely among schools that have more nutritional resources (OR = 6.74 and 5.23, respectively), participate in the BCSFVNP (OR = 4.54 and 2.71, respectively although only a trend towards statistical significance was observed for vegetable availability (p = .029)), and participate in the BC Milk Program (OR = 3.05 for fruit availability; however, no significant association was observed for vegetable availability). Associations with fruit and vegetable availability differed markedly among middle/high schools. Having more nutritional resources was the only school characteristic associated with vegetable availability in middle/high schools (OR = 5.78). In contrast, no school characteristics were significantly associated with fruit availability in middle/high schools. In addition, none of the school characteristics were associated with availability of pizza/hamburgers/hot dogs in elementary and middle/high schools. Finally, middle/high schools that have healthier nutritional practices (i.e., which align with upcoming provincial/state guidelines) were less likely to have the following food/beverage items available at school chocolate candy (OR = .80), sugar-sweetened beverages (OR = .77), and french fried potatoes (OR = .80, although only a trend towards statistical significance was observed p = .019).

### Associations with school internal and external influences

School internal and external influences were not associated with availability of any food/beverage items examined in this study.

## Discussion

Understanding environmental factors associated with the availability of specific food/beverage items at school is an important first step to ensure students have the opportunity to make healthy nutritional decisions at school. As previously found, the availability of food and beverages was much lower in elementary schools than in middle/high schools [3-5]. Overall, three school environmental factors were associated with the availability of specific food/beverage items at schools: (1) having more nutritional resources, (2) participation in provincial/state nutritional programs, and (3) having nutritional practices that align with upcoming mandated provincial/state nutritional guidelines. Associations among these environmental factors with availability of specific food/beverage items were complex as they varied by the type of food/beverage items examined and differed by grade.

We found that fruit and vegetable availability was significantly higher in elementary schools that have more nutritional resources. To better understand these findings, it is important to highlight the nutritional context of Canadian schools in BC. Unlike other countries, Canada does not have a federally mandated school meal/breakfast program [32]. As a result, many elementary schools in BC lack the amenities to refrigerate and store fresh fruit and vegetables and to prepare (cook or reheat) school meals. This partly explains why the availability of fruit and vegetables in BC elementary schools were found to be much lower than U.S. elementary schools (27% versus 68%, respectively [4]). In the context of BC, our findings might highlight the need to equip elementary schools with an appropriate refrigeration system to enable them to provide more fruit and vegetables to their students. Furthermore, fruit and vegetable availability may be limited to being available as snacks only as many of the permanent food outlets (i.e., school stores, cafeteria, or vending machines) in elementary schools are not preparing meals. However, many elementary schools (82%) have external vendors bringing A La Carte school lunch options (e.g., pizza, hamburgers, and hot dogs) at varied frequencies (e.g., multiple times a week to once a month) [3]. Potentially, fruit and vegetable availability might be increased by having external vendors change their offerings which could be achieved through policy strategies or incentive programs. In addition, nutritional resources were found to influence availability of food in middle/high schools; however, only an association with vegetable availability was observed. While food and beverages are more widely available in middle/high schools [3], there are still a large number of schools that do not offer lunch options. This might explain why we observed an association between vegetable availability and school nutrition resources in middle/high schools. Unlike elementary schools, middle/high schools reported greater availability of fruit at schools which may explain why we did not find an association between fruit availability and nutritional resources in these schools.

We found that participation in the BCSFVNP was associated with fruit availability in elementary schools. These findings may suggest that fruit availability in elementary schools is a result of participating in the BCSFVNP. If this were the case, it would not reflect the broader availability of fruit on a daily basis since the BCSFVNP does not provide enough servings of fruit and vegetables to meet the required daily for each student. While the intent of the program is to encourage students to eat fresh fruit and vegetables, participation in such programs while important, is not enough to ensure that students eat fresh fruit and vegetables every day while at school. Alternatively, fruit availability in elementary schools might be higher in schools that participate in BCSFVNP as they have greater capability to store fresh fruit snacks. As the nutritional context of elementary schools in BC may be better equipped to provide fruit and vegetables as snacks rather than integrating them into the lunch meal (as lunch meals are often brought by external vendors), it might explain why we did not find an association with vegetables as fresh fruit snacks might be easier to sell since they require little or no preparation. Lack of significant associations in middle/high schools may have resulted since participation in the BCSFVNP is much lower in these schools. Although the program is equally available to all grades, more elementary schools than middle/high schools participate in this program (43% versus 26%, respectively). Participation in the program is free; however, it requires schools to identify a volunteer to administer and manage the distribution of these food every other week. Therefore, participation might be easier to manage in elementary schools, as these schools are typically smaller and have less complex schedules. Finally, the difference in associations between elementary and middle/high schools may be reflective of the fact that the availability of all food and beverages in middle/high schools is markedly higher than in elementary schools [3]. Furthermore, we found that elementary schools participating in the BC Milk Program had more fruit availability. This finding can be explained by the nutrition environment of elementary schools in BC. Participation in the BC Milk Program requires schools to have an appropriate refrigeration system. Once they are equipped with such a system, they are in a better position to store food items. Again, finding an association with fruit might reflect that it is easier to sell fruit as snacks compared to vegetables. Freshly prepared vegetables require more preparation and appropriate storage while many whole fruit do not. Fresh fruit snacks may also be more appealing than vegetable snacks and are, therefore, more likely to be purchased. This association was only observed in elementary schools and may be explained by the fact that middle/high schools participate less in the program (38% versus 26%, respectively).

Finally, we found that middle/high schools that have healthier nutritional practices aligning with upcoming mandated provincial/state guidelines were less likely to have chocolate candy, sugar-sweetened beverages, and french fried potatoes (although the latter was a trend). Our data was collected 6-months prior to the deadline at which BC schools were expected to comply with the mandated guidelines introduced in 2005. While we do not know whether the schools have changed their environment as a result of the mandated guidelines, limiting availability of less healthful food and beverages through policy change has been associated with improved dietary intake in students [42-44]. As policies are increasingly being used to modify the school environment, it is important to assess the extent to which schools have implemented these guidelines/policies as intended to ensure decreased availability of less healthful food and beverages in schools.

The findings of this study should be interpreted in light of the limitations of the study. Firstly, associations were examined in a cross-sectional sample which precludes us from identifying factors that predict availability of food and beverages at schools. Secondly, we did not examine the extent to which the school environmental factors influenced dietary intake as this study focussed on availability as an important first step in ensuring a healthier nutritional environment. Thirdly, all measures were assessed with self-report which is known to be associated with a number of limitations. As many of the measures were developed or adapted for this study, we have limited information about the validity of these measures and whether the constructs we used were optimally operationalized. We evaluated the psychometric properties of these measures to improve the validity of our findings; however, future studies should further examine the properties of these measures. Furthermore, the availability of food and beverages was measured with an established measure [40]; however, the measure does not distinguish whether or not healthier versions were served. As schools are increasingly encouraged to provide healthier versions of less healthful food (e.g., pizza, hamburgers, and hot dogs), future studies are encouraged to incorporate this distinction in their measurement. Lastly, the food environment in public schools in Canada can vary greatly by province/state as policies or mandated guidelines are primarily set at the provincial/state level. Therefore, our findings may not be generalized to other provinces/states and countries with different structures governing the school food environment.

Students have widespread access to less healthful food and beverages at school. Therefore, there is strong support for developing school food policies/guidelines to influence the school environment. The extent to which schools can implement mandated policies/guidelines will depend to a certain extent upon factors within the school environment. This study found three environmental factors were associated with the availability of specific food/beverage items at school: having more nutritional resources, participation in provincial/state nutritional programs, and having nutritional practices that align with upcoming mandated provincial/state nutritional guidelines. As school policies/guidelines are increasingly being considered to modify the eating behavior of children at school, it is important to gain a better understanding of the factors associated with availability of certain food/beverage items at school to better understand factors that may facilitate attempts to change the school food environment.

## Competing interests

None of the authors have any competing interests.

## Author’s contributions

LCM contributed to the design, data collection, data analyses, data interpretation, the drafting of the paper, and main writing to the paper. JEdN contributed to the data analyses, data interpretation, and drafting sections of the paper. All authors have read and approved the final manuscript.
